# Successful Treatment of Catatonia: A Case Report and Review of Treatment

**DOI:** 10.7759/cureus.26328

**Published:** 2022-06-25

**Authors:** Kevin Malone, Sall Saveen, Christopher M Stevens, Shawn McNeil

**Affiliations:** 1 Biomedical Engineering, Louisiana State University Health Sciences Center, Shreveport, USA; 2 Psychiatry, Louisiana State University Health Sciences Center, Shreveport, USA; 3 Interventional Radiology, Louisiana State University Health Sciences Center, Shreveport, USA

**Keywords:** antipsychotic medication, electroconvulsive therapy (ect), lorazepam challenge test, benzodiazepines, bush-francis catatonia rating scale, catatonia

## Abstract

Herein, we report the case of a 20-year-old Caucasian male with a previous psychiatric history of schizophreniform, autism, unspecified intellectual disorder, and past medical history of hypertension, who presented after a suicidal attempt. One month prior to admission for the suicidal attempt, the patient had mutism. While admitted, the patient showed signs of mutism, posturing, negativism, and waxy flexibility. Treatment with both aripiprazole and lorazepam was effective and reversed the patient’s catatonia after low-dose titration. This case highlights the importance of reviewing patient history and presenting symptoms in the management of catatonia. Additionally, this case provides an opportunity to review the diagnostic approach and treatment type used for patients presenting with catatonia.

## Introduction

The term catatonia is a derivation from the Greek words kata (down) and tonas (tension or tone) [1]. Catatonia was first described by Dr. Karl Kahlbaum, a German psychiatrist, in 1874. His description of catatonia focused heavily on the phenomenology of catatonia and the course of the illness [2]. His work, which he presented in the late 1800s, supported the notion that catatonia resulted from multiple causes and was not an independent disease entity [2]. Many clinicians matched their understanding of catatonia with that of Kahlbaum’s understanding recognizing catatonia as a multicausality phenomenon [2].

In more recent years, catatonia has been defined as a clinical syndrome marked by at least three of the following 12 psychomotor symptoms outlined in the DSM-5: catalepsy, waxy flexibility, stupor, agitation, mutism, negativism, posturing, mannerisms, stereotypies, grimacing, echolalia, or echopraxia. Catatonia’s primary clinical feature is a marked psychomotor disturbance with symptoms ranging from stupor to agitation [3]. Around 90,000 cases of catatonia occur in the United States per year [4]. Catatonia has a reported prevalence of 7% to 38% in psychiatric patients [5]. People with mood disorders, psychosis, increased age, increased frequency of depressive episodes, and cognitive impairment are at an increased risk for developing catatonia [5-7].

We report a case of catatonia in a 20-year-old Caucasian male. Treatment with Aripiprazole and Lorazepam was effective and resulted in the reversal of the patient’s catatonia after low dose titration. This case highlights the management of catatonia, the importance of patient history, and presenting symptoms. Additionally, this case provides an opportunity to review the diagnostic approaches and treatment types that are used for patients presenting with catatonia-like symptoms.

## Case presentation

A 20-year-old Caucasian male with a previous psychiatric history of schizophreniform, autism, an unspecified intellectual disorder, and a past medical history of hypertension, presented to the emergency room for a “syncope” or “seizure-like” activity. The patient had no prior history of seizures or syncope.

Approximately two months prior, the patient went on a camping trip where the patient admitted to using marijuana and possibly some other unknown substances. After returning from a camping trip the patient stated he had several hallucinations and stated, “I am not smoking weed anymore.” The patient stated at the time he was hearing voices, claiming that he was possessed by a demon, and admitted to both suicidal and homicidal ideation. For the next month, the patient became withdrawn from social contacts demonstrating increased paranoia and disturbances in speech.

The patient was seen pacing the floor inside the family’s residence with a gun which the mother had to wrestle out of his hands. Additionally, the patient was seen to have cuts on his neck. The patient was admitted to a behavioral unit after being evaluated in the ED. Prior medication history was unknown other than quetiapine use in the past of unknown dose or frequency. The patient was initially started on paliperidone 3 mg twice a day and haloperidol 1 mg intramuscular twice a day as needed for agitation. His medication regime was later changed to paliperidone 12 mg in the evening alone. He was then started on a long-acting paliperidone injection of 234 mg before discharge. The patient was discharged home two weeks after admission to the behavioral unit with a follow-up appointment at a psychiatry clinic which the patient failed to attend.

The mother states that after the missed appointment the patient said he wanted to kill himself and rushed out of his residence to look for a hidden gun at his uncle's house. The patient was detained and told that he needed to be admitted to the hospital for suicidal ideations. The patient’s mother reported he had a single isolated “seizure-like” incident that lasted two minutes. The patient experienced what was described as a loss of consciousness, body movements, and no discernible post-ictal period. The seizure was witnessed by the family. The patient was brought to the emergency department, where he endorsed suicidal ideations and hopelessness. After being medically cleared, the patient was admitted to a behavioral unit.

On presentation to the behavioral unit, the patient is catatonic and either unwilling to speak to the interviewer or unable to. Bush-Francis Catatonia Rating Scale was 24 on presentation. The patient was admitted to the inpatient unit and was placed on appropriate precautions. The patient was started on paliperidone 6 mg daily, lorazepam 0.5 mg twice a day, and sertraline 100 mg daily. The patient stayed in bed and did not move. The patient refused to get out of bed and would not respond to stimulation. The patient refused oral medications and was placed on forced medication protocol. On the third day after admission, the patient remained catatonic-like and lorazepam was increased to 1 mg twice a day, paliperidone was discontinued and aripiprazole was started at 10 mg daily. On the fifth day of admission, the patient began to get out of bed and seldomly walked in the halls. He was noted to seem to become frightened at unknown entities in the room; aripiprazole was increased to 15 mg daily. On the seventh day, the patient was noted to have some improvements in affect, the patient was seen walking with a shuffling gait, and no swing of arms. The dose of aripiprazole was increased to 20 mg daily. The next day lorazepam dose frequency was increased to 1 mg three times a day. The patient had significant improvement in affect, seldomly spoke, and was now walking out of the room more often than not. On the ninth day no medication changes were made, the patient states he is doing well and is smiling at staff. The patient is less stiff, with a swing of arms, and no longer has a shuffling gait. The next day the patient was deemed to be psychiatrically stable. Bush-Francis Catatonia Rating Scale was 0. The patient was in good spirits, answering questions and verbalizing understanding of the plan moving forward. After discharge, the patient continued to improve and has not had any readmissions after six months (Table 1).

**Table 1:** Timeline of patient’s stay in the behavioral hospital.

Timeline Regarding the Most Recent Admission	Events
2 months prior	The patient went on a camping trip using marijuana and possibly some other substances. After returning home the patient had several hallucinations, hearing voices, claiming that he was possessed by a demon.
The next month	The patient became withdrawn from social contacts, paranoid and decreased speaking for a month.
1 month prior	The patient was found pacing the floor inside the family’s residence with a gun and cuts on his neck. The patient was admitted to a behavioral unit and initially started on paliperidone 3 mg twice a day and haloperidol 1 mg intramuscular twice a day. His medication regime was later changed to paliperidone 12 mg in the evening alone. He was then started on a long-acting paliperidone injection of 234 mg before discharge home after a two week admission.
2 weeks before	The patient fails to follow-up to his psychiatry appointment.
Day of admission	The patient states that he wanted to kill himself and rushed out of his residence to look for a hidden gun, was detained and told that he needed to be admitted. He then proceeded to have a single isolated “seizure-like” incident which was described as a loss of consciousness, body movements, and no discernible post-ictal period. After being medically cleared, the patient was admitted to a behavioral unit. Bush-Francis Catatonia Rating Scale 24.
Admission day 1	The patient is catatonic and either unwilling to speak to the interviewer or unable to. Started on paliperidone 6 mg daily, lorazepam 0.5 mg twice a day, and sertraline 100 mg daily. Patient refused to get out of bed and would not respond to stimulation. The patient refused oral medications and was placed on forced medication protocol.
Day 3	The patient remained catatonic-like and lorazepam was increased to 1 mg twice a day, paliperidone was discontinued and aripiprazole was started at 10mg daily.
Day 5	The patient began to get out of bed, and seldomly walked in halls. He was noted to seem to become frightened at unknown entities in the room. Aripiprazole was increased to 15 mg daily.
Day 7	The patient was noted to have some improvements in affect, the patient was seen walking with shuffling gait, and no swing of arms. Dose of aripiprazole was increased to 20 mg daily.
Day 8	Lorazepam dose frequency was increased to 1 mg three times a day. The patient had significant improvement of affect, seldomly spoke, and was now walking out the room more often than not.
Day 9	No medication changes were made, the patient states he is doing well and is smiling at staff. The patient is less stiff, with swing of arms, and no longer a shuffling gait.
Day 10	The patient was deemed to be psychiatrically stable. Bush-Francis Catatonia Rating Scale of 0. Patient was in good spirits, answering questions and verbalized understanding of the plan moving forward.
The next six months	After discharge, the patient continued to improve and has not had any readmissions after six months.

## Discussion

Diagnostic approach

The Bush-Francis Catatonia Rating Scale (BFCRS), designed in 1996 by Bush et al., is currently the most preferred method for diagnosing catatonia due to its five-minute administration time, reliability, and validity [8,9]. Two versions of the BFCRS exist, an extended one, which consists of 23 items rated on a scale from 0 to 3, and a shortened one consisting of only the first 14 items expressed in the extended version [10]. Other scales used to diagnose catatonia include the Modified Rogers Catatonia Scale, Rogers Catatonia Scale, a revision of the BFCRS proposed by Ungvari, Northoff Catatonia Rating Scale, Braunig Catatonia Rating Scale, and the Kanner Scale [10,11]. No laboratory tests that define catatonia exist [11].

Benzodiazepines are the mainstay of the treatment of catatonia but are also commonly used as a diagnostic probe [12]. During the test, the patient is examined for signs of catatonia, and 1 or 2 mg of Lorazepam is administered intravenously. After a five-minute period, the patient is then re-examined. If there has been no change, a second dose is given, and the patient is again reassessed. A positive response is a marked reduction by at least 50% of catatonic signs and symptoms, as measured with the BFCRS. Favorable responses usually occur within 10 minutes of administration [13,14].

Review of treatment

Regardless of the underlying diagnosis, benzodiazepines are generally accepted as the first line of treatment. Specifically, lorazepam is the first-choice drug because it demonstrates the highest frequency of use and a nearly 80% remission rate. The successful use of other benzodiazepines, including diazepam, oxazepam, or clonazepam, has also been reported [12]. Studies indicate that children and adolescents appear to show a better response to benzodiazepines than adults. For example, in a study of hospitalized patients with catatonia who were between the ages of 9 and 19, benzodiazepines were found to improve catatonia symptoms in 65% of cases, with no relation between dose and improvement [15]. Additionally, a five-year follow-up study indicated that chronic catatonia associated with schizophrenia has less dose-response to benzodiazepines [12]. In 2019, Cochrane attempted to review randomized control trials of head‐to‐head comparisons of benzodiazepine, other drugs, placebo, or electroconvulsive therapy (ECT); only one trial was found that compared two benzodiazepines (lorazepam vs oxazepam) and found no clear difference between these two treatments [16]. For the treatment of catatonia, the typical starting dose is 1 or 2 mg of lorazepam every four to 12 hours, and adjusting the dose to relieve catatonia without causing sedation [17]. An adequate response is usually seen within three to seven days. There is no consensus on how long benzodiazepines should be continued; however, it has been suggested to trial discontinuation after the underlying psychological disorder has resolved [18].

All prescribed medications should be carefully evaluated for their potential to induce catatonic symptoms [12]. This is especially true in relation to antipsychotic medications. It is unclear the role of antipsychotic medication in catatonia; however, it is generally encouraged to discontinue antipsychotic treatment in patients presenting with catatonia on current antipsychotic medications [13]. Both first-generation and second-generation antipsychotics may result in maintaining or worsening a patient’s catatonic state. The risk of worsening catatonia appears to increase because of a larger D2-blockade [12].

The role of second-generation antipsychotics in the treatment of catatonia is unclear as past research has mostly focused on cases with schizophrenia [12]. Second-generation antipsychotics have weak GABA-agonist activity and 5HT2-antagonist activity that could help stimulate dopamine release in the prefrontal cortex and thus may alleviate catatonic symptoms [17]. Several papers have reported on the beneficial effect of second-generation antipsychotics, such as clozapine, olanzapine, risperidone, and quetiapine [12,19-21].

Once treatment with benzodiazepines or ECT is started and there is a reduction in catatonic features, there may be a role for second-generation antipsychotics for patients with schizophrenia, especially to target psychotic symptoms, such as delusions or hallucinations [12]. However, the use of second-generation antipsychotics in the presence of catatonia should be evaluated for each case [12].

In the past, amantadine and its derivative, memantine, have been used as a treatment for catatonia. In one report, 64% of patients experienced substantial improvement (N=25). Another case reported a rapid reduction of catatonic symptoms in a 68-year-old patient after the administration of memantine [22]. Although there have been other reports of similar improvement using amantadine and memantine, they are not commonly used in practice, as this is still an ongoing area of research [12].

ECT should be started in patients with catatonia who are not responding to benzodiazepines or when a rapid response is required in severe cases or for life-threatening conditions; specifically, there is literature to support the initiation of ECT "without delay" in the setting of inadequate response to benzodiazepines [12]. Despite the absence of randomized controlled evidence, the efficacy of ECT in treating catatonia is generally acknowledged. Only one randomized controlled trial of ECT has been published, which showed a significant reduction in catatonic symptoms in the ECT group as compared to the control group [12]. While the efficacy of ECT has not been systematically studied, some small studies and hundreds of case reports have described its success [12]. ECT may be more effective in younger patients or those experiencing autonomic dysregulation, particularly increased body temperature [23]. Further, it is generally advised to stop using psychopharmacological agents prior to the initiation of ECT [12].

## Conclusions

In this manuscript, we presented a case of catatonia in a 20-year-old Caucasian male in which treatment with Aripiprazole and Lorazepam was effective in reversing the patient's symptoms. In addition, an examination of the current diagnostic approach and treatment type used for patients presenting with catatonia was conducted. We found that the BFCRS is currently the most preferred method for diagnosing catatonia. We also concluded that benzodiazepines, despite the successful use of other pharmacological agents, are the most popular treatment source used for catatonia patients, with Lorazepam commonly being the initial choice of treatment. Second-generation antipsychotic medications may be used on a case-by-case basis and provide some benefits. For patients who are not responding to benzodiazepines or when a rapid response is required in severe cases or life-threatening conditions, ECT should be initiated.
